# The Effect of a Screening and Treatment Program for the Prevention of Fractures in Older Women: A Randomized Pragmatic Trial

**DOI:** 10.1002/jbmr.3815

**Published:** 2019-08-01

**Authors:** Thomas Merlijn, Karin MA Swart, Natasja M van Schoor, Martijn W Heymans, Babette C van der Zwaard, Amber A van der Heijden, Femke Rutters, Paul Lips, Henriëtte E van der Horst, Christy Niemeijer, J Coen Netelenbos, Petra JM Elders

**Affiliations:** ^1^ Amsterdam UMC, Vrije Universiteit Amsterdam, General Practice and Elderly Care Medicine Amsterdam Public Health Research Institute Amsterdam Netherlands; ^2^ Stichting Artsen Laboratorium en Trombosedienst, Koog aan de Zaan Netherlands; ^3^ Amsterdam UMC, Vrije Universiteit Amsterdam, Epidemiology and Biostatistics Amsterdam Public Health Research Institute Amsterdam Netherlands; ^4^ Jeroen Bosch Ziekenhuis, Department of Orthopaedics, ’s‐Hertogenbosch Netherlands; ^5^ Amsterdam UMC, Vrije Universiteit Amsterdam, Internal Medicine, Endocrine Section Amsterdam Netherlands

**Keywords:** FRACTURE PREVENTION, FRACTURE RISK ASSESSMENT, SCREENING, CLINICAL TRIALS

## Abstract

Population screening for fracture risk may reduce the fracture incidence. In this randomized pragmatic trial, the SALT Osteoporosis Study (SOS), we studied whether screening for fracture risk and subsequent treatment in primary care can reduce fractures compared with usual care. A total of 11,032 women aged 65 to 90 years with ≥1 clinical risk factor for fractures were individually randomized to screening (*n* = 5575) or usual care (*n* = 5457). Participants in the screening group underwent a screening program, including bone densitometry and vertebral fracture assessment. Participants with a high 10‐year fracture probability (FRAX) or a vertebral fracture were offered treatment with anti‐osteoporosis medication by their general practitioner. Incident fractures as reported by questionnaires were verified with medical records. Follow‐up was completed by 94% of the participants (mean follow‐up = 3.7 years). Of the 5575 participants in the screening group, 1417 (25.4%) had an indication for anti‐osteoporosis medication. Screening and subsequent treatment had no statistically significant effect on the primary outcome fracture (hazard ratio [HR] = 0.97; 95% confidence interval [CI] 0.87–1.08), nor on the secondary outcomes osteoporotic fractures (HR = 0.91; 95% CI 0.81–1.03), major osteoporotic fractures (HR = 0.91; 95% CI 0.80–1.04), hip fractures (HR = 0.91; 95% CI 0.71–1.15), falls (odds ratio [OR] = 0.91; 95% CI 0.72–1.15), or mortality (HR = 1.03; 95% CI 0.91–1.17). Post hoc explorative finding suggested that screening might be most effective after a recent fracture (HR = 0.65; 95% CI 0.44–0.96 for major osteoporotic fractures and HR = 0.38; 95% CI 0.18–0.79 for hip fractures). The results of this study might have been compromised by nonparticipation and medication nonadherence in the screening group. Overall, this study does not provide sufficient indications to consider screening for fracture prevention. However, we cannot exclude its clinical relevance to reduce (major) osteoporotic fractures and hip fractures because of the relatively small number of women with a treatment indication in the intervention group. © 2019 The Authors. *Journal of Bone and Mineral Research* Published by Wiley Periodicals, Inc.

## Introduction

Fractures are an age‐related problem that may lead to disability and decline in functioning, especially at older age.^1^, ^2^, ^3^, ^4^, ^5^ Moreover, fractures are associated with an increased mortality rate for many years after the fracture.^6^ In the Netherlands, in 2010 more than 110,000 fractures occurred in persons aged ≥50 years, and the incidence and direct health care costs are expected to increase 40% and 50%, respectively, in 2030.^7^ Hence, there is a need to evaluate strategies that might reduce fracture incidence.

Fracture prevention strategies will succeed if persons at risk are identified and if effective interventions are available that are acceptable to patients. However, identification of persons at risk for fractures is a challenge. Although osteoporosis is defined as a low bone mineral density (BMD) measured with bone densitometry, BMD only partially accounts for fracture risk,^8^ and therefore bone densitometry alone is not appropriate as a screening instrument. Other factors such as falling, previous fractures, or a positive family history of hip fractures are important as well. In the last decade, the added value of clinical risk factors has been recognized in fracture risk prediction. These factors in addition to bone densitometry have been shown to increase the accuracy of the prediction of fractures and can be used to identify patients at high risk for fractures.^(9)^

As effective anti‐osteoporosis medication is available,^10^, ^11^ population screening for fracture prevention has come within reach. Screening in two recent randomized trials, the Risk‐Stratified Osteoporosis Strategy Evaluation (ROSE) study and the Screening in the Community to Reduce Fractures in Older Women (SCOOP) study, did not significantly reduce the incidence of (osteoporotic) fractures, but secondary analyses showed a reduction of hip fractures.^12^, ^13^ The current randomized pragmatic study started in the same period as these studies and could provide a final answer as to whether screening for fracture prevention is effective. This study aimed to examine whether screening for fracture risk and subsequent treatment in primary care can reduce fractures in comparison to usual care.

## Materials and Methods

The SALT Osteoporosis Study (SOS) is a pragmatic randomized controlled trial performed in the Netherlands. Details of the methods have been published.^(14)^

Women aged 65 to 90 years from the registries of participating general practitioners (GPs) were invited for participation. Women were included if they had ≥1 clinical risk factor for fractures, as assessed with a baseline questionnaire: a previous fracture after age 50 years, a parental hip fracture, low body weight (body mass index [BMI] <19 kg/m^2^), rheumatoid arthritis, early menopause (<45 years of age), malabsorption syndrome, chronic liver disease, type I diabetes mellitus, or immobility (severe walking difficulties and/or use of walking aid). Exclusion criteria were a short life expectancy according to the GP, current use of anti‐osteoporosis medication or in preceding 5 years, recent densitometry, terminal illness, body weight >135 kg, or corticosteroid use ≥7.5 mg prednisone equivalent/day. Women were either excluded by their GP or by using the information on the questionnaire.

Eligible women were randomized, using a parallel‐group design (1:1), to the screening or usual care group. The randomization sequence was computer‐generated and group allocation was automatically assigned to every new included participant via our web‐based database. Participants in the screening group were invited to participate in a screening program. Subsequently, participants with a treatment indication for anti‐osteoporosis medication received a treatment program. After 18 and at least 36 months, follow‐up questionnaires were completed. Complete blinding of participants and study team was not feasible. Nevertheless, processing questionnaires and verification of fractures were performed blinded.

Participants were recruited from July 2010 to April 2014. The study ended in July 2017. All participants gave written informed consent. The study complies with the Declaration of Helsinki. The trial was approved by the Dutch Health Council (2009/05WBO), and a license was provided by the Dutch Ministry of Health, Wellbeing and Sports (PG/)GZ‐2.978.265).

### Screening

Screening consisted of dual‐energy X‐ray absorptiometry (DXA), vertebral fracture assessment (VFA), risk factor evaluation for fractures (FRAX) and falls, and blood tests to exclude secondary osteoporosis.^14^

DXA measurements were performed using a Hologic Discovery device under standardized procedures. Lateral images of the spine were made using VFA. All images were evaluated by two trained independent observers; in case of a mismatch, there was a final judgment by a third observer. Our method was based on the semiqualitative method of Genant. First, deformities were judged as fracture or not, mainly based on endplate depression. Second, height loss was estimated according to the grading of Genant.^(15)^ Only vertebrae with a height reduction ≥20% in the lumbar spine or ≥25% in the thoracic spine were included as vertebral fracture.

Details on falling were assessed with the baseline questionnaire. An increased fall risk was defined as two or more falls in the previous year or one fall combined with a reduced mobility or fear of falling.^14^

Because a Dutch FRAX tool was not available at the start of the study, the UK version of the FRAX was used with cut‐off values derived from the data from a representative sample of Dutch older persons (Table 1).^14^, ^16^ Age‐dependent treatment cut‐offs were applied to avoid counterintuitive clinical practice whereby almost all participants of 80 years and older would have an indication for anti‐osteoporosis medication at low treatment thresholds and only few participants of 70 years and younger at higher thresholds. Ten‐year major osteoporotic fracture probability according to a FRAX‐BMD score above age‐dependent thresholds in combination with a DXA *T*‐score ≤−2 or a prevalent vertebral fracture was an indication for treatment with anti‐osteoporosis medication.^14^ In addition, the treatment indication according to the actual Dutch GP guideline was followed.^17^

**Table 1:** FRAX Thresholds for Treatment, Stratified for Age

Age (years)	FRAX treatment threshold
65–69	>15%
70–74	>18%
75–79	>24%
80–84	>28%
85–91	>32%

FRAX = fracture risk assessment tool.

### Treatment

Most GPs attended a group education session on general aspects of osteoporosis and its treatment.^14^ All GP practices were visited to receive instructions about the protocol and the treatment program. For each participant with an indication for treatment in the screening group, the GP received a personalized treatment advice, formulated by an expert team of experienced GPs. The GP could contact the expert team for further advice if needed. First‐choice anti‐osteoporosis medication was alendronic acid 70 mg/week or risedronic acid 35 mg/week. In addition, the experts formulated personalized advices on calcium and vitamin D supplementation, notification of a high fall risk, additional diagnostics, or referral to secondary care. GPs were allowed to deviate from the protocol using their professional insight.^14^ As specified by the Dutch GP guideline, GPs were instructed to organize consultations to optimize adherence and to evaluate side effects.^17^ GPs were therefore offered an option to use an application for consult notification. Self‐reported medication adherence was assessed on the follow‐up questionnaires.

### Usual care

**Figure 1:**
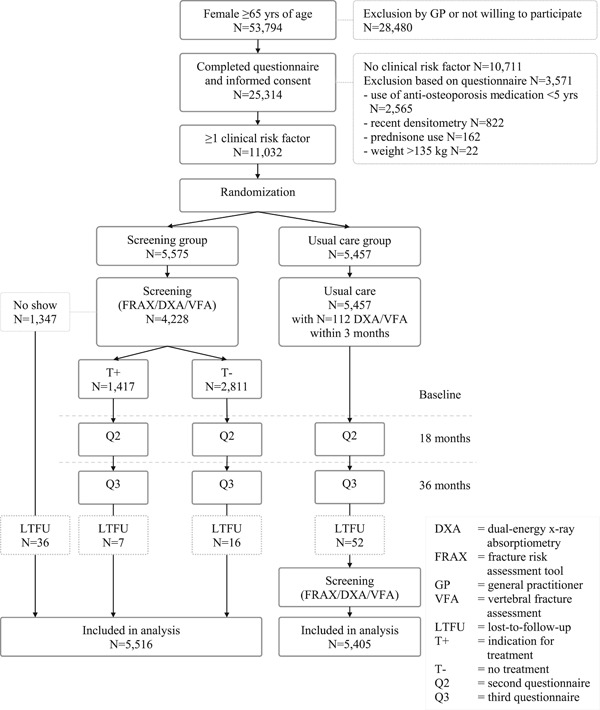
Flow chart of the SALT Osteoporosis Study.

A waiting list construction was applied to the usual care group in which participants were offered the same screening program as the screening group after study completion (Fig. 1). For ethical reasons, this could not be done at baseline without informing participants about the results. At baseline, participants in the usual care group with an indication for DXA and VFA, according to the Dutch GP guideline, were notified accordingly and were advised to contact their GP as part of usual care.^14^

### Outcomes

The primary outcome was any type of fractures, and secondary outcomes were osteoporotic fractures, hip fractures, falls, and death. Post hoc, we added major osteoporotic fractures to be able to compare the findings with other studies. Furthermore, predefined evaluation of costs was left out as outcome because of the lack of funding for this evaluation.

Self‐reported fractures on the follow‐up questionnaires were verified with the GP or hospital medical record. Moreover, we tried to trace all participants who did not complete the questionnaires in their GP registries for fractures and death. Osteoporotic fractures were defined as all fractures except for skull, finger, hand, toe, and foot fractures.^14^ Major osteoporotic fractures were defined as all hip, vertebral, wrist, and humerus fractures.^12^ Falls in the past year were self‐reported on the follow‐up questionnaires. Death reported by relatives was registered during the trial.

### Statistical analyses

The sample size calculation determined the number of participants in the high‐risk group. Assuming that fractures among screened high‐risk participants would be reduced by 35%,^10^, ^11^ that 3% of the usual care group would receive treatment, and that 30% would be lost to follow‐up, while aiming for a β of 0.2 and α of 0.05, we calculated that 1700 high‐risk participants with an indication for treatment per group would be needed. We further assumed that 50% of the screened participants would have a treatment indication, resulting in a total sample size of 3400 participants per group. Because the proportion of the screened participants with an indication for treatment turned out to be 33% instead of the assumed pilot‐based 50%, we included more participants than the anticipated 3400 per group.

Differences between the screening and usual care group were tested by *t* tests, Mann–Whitney *U* tests, or chi‐square tests. In addition, differences between participants in the screening group with and without screening results were tested. Using an intention‐to‐treat approach in which all randomized participants were analyzed irrespective of screening completion, Kaplan–Meier and Cox proportional hazard models were used to study the effect on time to first fracture. Cox models were adjusted for baseline variables that differed between groups and repeated for the secondary outcomes. Interaction effects were tested for age and previous fractures by adding interaction terms with group allocation to the model. In exploratory post hoc analyses, interaction with a recent fracture (<2 years before baseline) was examined as well. A *p* value for interaction of <0.10 was considered as justification for stratified analyses. In all other analyses, *p* < 0.05 was considered statistically significant. Analyses were performed using IBM SPSS Statistics 22 (IBM Corp., Armonk, NY, USA).

## Results

### Participation details

In total, 217 GP practices participated. Fig. 1 shows that 25,314 (47.1%) of 53,794 invited participants completed the initial questionnaire and that 11,032 participants were randomized. No significant differences between groups were observed at baseline (Table 2), except for a slightly higher proportion of participants in the screening group that consumed ≥3 glasses of alcohol/day.

**Table 2:** Baseline Characteristics of the Participants of SOS

	Intention‐to‐treat population		
	Usual care group (*n* = 5457)	Screening group (*n* = 5575)	Participants with a treatment indication in the screening group (*n* = 1417)
Age (years)	75.0 (6.8)	75.0 (6.7)	74.5 (6.5%)
Education level			
Low	1066 (20%)	1117 (20%)	237 (17%)
Intermediate	3650 (67%)	3711 (67%)	987 (70%)
High	631 (12%)	652 (12%)	174 (12%)
BMI (kg/m^2^)	26.7 (5.1)	26.8 (5)	25.2 (4.0)
Current smoking	675 (12%)	628 (11%)	169 (12%)
Alcohol use (≥3 glasses/d)	318 (6%)	380 (7%)	104 (7%)
Calcium supplement use	1173 (21%)	1166 (21%)	327 (23%)
Vitamin D supplement use	1131 (21%)	1136 (20%)	318 (22%)
No. of medicines	3 [1–6]	3 [1–6]	2 [1–5]
Poor mobility	1698 (31%)	1667 (30%)	285 (20%)
Use of walking aid	1786 (33%)	1829 (33%)	328 (23%)
Fallen in past year	1717 (31%)	1763 (32%)	437 (31%)
Fracture after age 50 years	2340 (43%)	2449 (44%)	836 (59%)
Hip fracture in family	1745 (32%)	1771 (32%)	592 (42%)
Early menopause (<45 years of age)	720 (13%)	758 (14%)	191 (13%)
Corticosteroid use (5–7.5 mg/d)^a^	27 (<1%)	30 (<1%)	7 (<1%)
Diabetes type I	66 (1%)	63 (1%)	12 (1%)
Rheumatoid arthritis^a^	101 (2%)	122 (2%)	32 (2%)
Malabsorption syndrome	128 (2%)	159 (3%)	40 (3%)
Chronic liver disease	31 (<1%)	25 (<1%)	5 (<1%)
10‐year major osteoporotic fracture probability (FRAX)	24.3 (10.5)	24.6 (10.8)	29.8 (11.9)
10‐year major osteoporotic fracture probability (FRAX‐BMD)	NA	16.8 (8.5)	23.9 (9.6)
10‐year hip fracture probability (FRAX)	11.3 (10.2)	11.6 (10.5)	15.7 (12.9)
10‐year hip fracture probability (FRAX‐BMD)	NA	5.8 (7.4)	10.6 (10.1)

SOS = SALT Osteoporosis Study; BMI = body mass index; FRAX = Fracture risk assessment tool; BMD = bone mineral density; NA = no BMD results available.

Data are presented as *n* (%), mean (SD), or median [interquartile range].

^a^ Verified in medical record.

Of 5575 participants in the screening group, 4228 (75.8%) participated in the screening program. On the one hand, participation was associated with a higher education level and being less frail but on the other hand with a history of fractures after the age of 50 years and hip fractures in the family. In the screening group, 1417 (33.5%) of 4228 participants who underwent the screening program had an indication for anti‐osteoporosis treatment. As part of usual care, 316 participants underwent DXA and VFA in the usual care group. One hundred twelve (35.4%) of 316 DXAs were performed within 3 months after baseline.

In the screening group, 982 (69.3%) of 1417 participants with a treatment indication reported to have started their medication, mainly bisphosphonates (96.2%). After 18 and 36 months, 842 (85.7%) of 982 and 657 (66.9%) of 982 starters, respectively, reported to still use anti‐osteoporosis medication. Among the participants in the screening group without a treatment indication, 31 (1.1%) of 2811 reported to use anti‐osteoporosis medication at 18 months and 68 (2.4%) of 2811 at 36 months. Of the participants who did not show up for screening, this was 25 (1.9%) of 1347 and 31 (2.3%) of 1347, respectively. In the usual care group, 167 (3.1%) of 5457 reported to use anti‐osteoporosis medication after 18 months and 214 (3.9%) of 5457 after 36 months. Overall, 1154 (20.7%) of 5575 participants in the screening group reported to have used anti‐osteoporosis medication during follow‐up versus 291 (5.3%) of 5457 participants in the usual care group.

Complete fracture follow‐up was available in 10,924 (99.0%) of 11,032 participants at 18 months and in 10,392 (94.2%) of 11,032 participants at the end of follow‐up. The follow‐up comprised 40,460.5 person‐years of observation.

### Effect of screening and subsequent treatment

In the screening group, 626 participants had a fracture (fracture rate = 3.1/100 person‐years) during follow‐up versus 632 participants in the usual care group (fracture rate = 3.2/100 person‐years). Time to first fracture was not significantly different between groups (log rank *p* value = 0.61, hazard ratio [HR] = 0.97; 95% confidence interval [CI] 0.87–1.08) (Table 2). No interaction effects with age (*p* = 0.60), a history of fracture after the age of 50 years (*p* = 0.48), or a recent fracture (<2 years before baseline, *p* = 0.34) were observed.

With respect to secondary outcomes, the HR of screening versus usual care was 0.91 (95% CI 0.81–1.03) for osteoporotic fractures, 0.91 (95% CI 0.80–1.04) for major osteoporotic fractures, 0.91 (95% CI 0.71–1.15) for hip fractures, and 1.03 (95% CI 0.91–1.17) for death (Table 3). The adjusted odds ratio (OR) for falling among participants with a high fall risk was 0.97 (95% CI 0.78–1.20) after 18 months and 0.91 (95% CI 0.72–1.15) after 36 months. Interaction effects of the intervention with a recent fracture were observed for the outcomes major osteoporotic fractures (*p* = 0.10) and hip fractures (*p* = 0.01). Explorative analyses revealed that among participants with a recent fracture (<2 years before baseline, screening *n* = 493 and usual care *n* = 473), significantly less major osteoporotic fractures (HR = 0.65; 95% CI 0.44–0.96, screening *n* = 43 versus usual care *n* = 60) and hip fractures (HR = 0.38; 95% CI 0.18–0.79, screening *n* = 10 versus usual care *n* = 25) occurred in the screening group compared with the usual care group.

**Table 3:** Cox Proportional Hazard Results of the Effectiveness of the Screening and Subsequent Treatment Program (Intention‐to‐Treat) on Fracture Risk and Mortality in SOS

	Screening group (*n* = 5516)		Usual care group (*n* = 5405)			
Outcome	Cases	Cases/100 person‐years	Cases	Cases/100 person‐years	Unadjusted hazard ratio (95% CI)	Adjusted hazard ratio (95% CI)^a^
All fractures	626	3.1	632	3.2	0.97	0.97
					(0.87–1.09)	(0.87–1.08)
Osteoporotic fractures	547	2.7	578	2.9	0.92	0.91
					(0.82–1.03)	(0.81–1.03)
Major osteoporotic fractures	427	2.1	452	2.3	0.92	0.91
					(0.81–1.05)	(0.80–1.04)
Hip fractures	133	0.7	143	0.7	0.91	0.91
					(0.72–1.15)	(0.71–1.15)
Mortality	499	2.5	479	2.4	1.02	1.03
					(0.90–1.16)	(0.91–1.17)

CI = confidence interval.

^a^ Adjusted for baseline alcohol use.

Among the participants in the screening group with a treatment indication, 164 osteoporotic fractures occurred (3.5/100 person‐years), 125 major osteoporotic fractures (2.4/100 person‐years), and 41 hip fractures (0.8/100 person‐years). Among participants who were still adherent with the treatment after 36 months, this was 67 (2.7/100 person‐years), 52 (2.1/100 person‐years), and 16 (0.6/100 person‐years), respectively. Because adherence was associated with lower age, higher education, less smoking, less mobility limitations, and less falling, these results should be interpreted with caution.

## Discussion

Our pragmatic screening and subsequent treatment program did not show a statistically significant reduction on fractures of any type, osteoporotic fractures, hip fractures, falls, or mortality in older women in primary care. However, the clinical relevance of the results cannot be excluded.

The reduction seems small compared with the effect of bisphosphonates on nonvertebral fractures as indicated by previous Cochrane meta‐analysis of experimental studies with a relative risk reduction of 20% to 53%, depending on bisphosphonates and fracture type.^10^, ^11^ However, there are important differences between the experimental medication trials and this pragmatic screening trial. First, the proportion of participants who received treatment in the intervention group is lower, namely basically all participants versus 1 of 4 participants. Second, in our usual care group, 5% of the participants also received treatment, whereas there is hardly any treatment contamination in placebo‐controlled studies. Moreover, the experimental studies did not have a pragmatic design and were performed in controlled study settings with measures to maintain a high medication adherence. The observed effects of the current screening program are the average effect over the study population, whereas the gain was achieved in the high‐risk group of screened participants who received treatment. Therefore, with the observed relative risk reductions of 9% in spite of dilution and contamination, the effect of screening on osteoporotic fractures and hip fractures could be considered clinically relevant.

For the success of this screening program, both the adherence to the screening program and the adherence to the treatment were important factors. However, 47% of the invited women consented to participate. Additionally, 25% of the participants assigned to our intervention group did not participate in the screening program, whereas these were the participants with the highest fracture risk. In addition, 31% of the participants with a treatment indication reported that they did not start taking the prescribed medication. At 18 months, only 57% of those with a treatment indication were actually using this medication, and at 36 months only 43%. The observed effects are therefore restricted by suboptimal participation and adherence.

The current findings were in line with the primary findings of the two previous randomized pragmatic screening trials for fracture prevention, the ROSE study and the SCOOP study. In the ROSE study (Denmark) that studied women aged 65 to 80 years, screening also did not statistically significantly reduce the incidence of the main outcome major osteoporotic fractures (HR = 0.91; 95% CI 0.83–1.01).^12^ Furthermore, in the SCOOP study (UK), performed among women aged 70 to 85 years, screening did not statistically significantly reduce the incidence of the main outcome osteoporotic fractures (HR = 0.94; 95% CI 0.85–1.03).^13^ Moreover, in the ROSE and the SCOOP studies, a clinically relevant effect on these primary outcomes cannot be excluded. Importantly, in both the ROSE and SCOOP studies, a statistically significant reduction in hip fractures was observed,^12^, ^13^ but we have some concerns about these significant secondary results of both the ROSE and SCOOP studies.

In the SCOOP study, the relative hip fracture reduction was 28%.^13^ This large effect raises attention, especially because it was not reflected by a substantial reduction of osteoporotic fractures in SCOOP and only small effects were to be expected: in SCOOP only 11% of the intervention group was treated with anti‐osteoporosis medication. Simple calculation shows that if the effect was only attributable to treatment in the intervention group, then almost all hip fractures (86%) in that group would have been prevented.^18^ Although the SCOOP design seems robust and not vulnerable to bias, further evaluation is needed, for instance, evaluation of reporting bias or consideration of screening effects unrelated to medication use.

Second, the additional post hoc analysis that showed a hip fracture reduction in the ROSE study is prone to bias. In that analysis, a subgroup of participants in the intervention group was compared with a subgroup of participants in the control group.^12^ However, participants in the intervention group not interested in the DXA (12%) and DXA dropouts (17%) were left out, resulting in a younger and healthier selection in the intervention group.^12^ In the SOS, DXA participation was associated with a higher education level, being less frail, a history of fractures, and hip fractures in the family. The observed effect in ROSE might be explained by this selection. To ground this assumption, we applied the same selection to our population, leaving out the participants in the intervention group without DXA results (no shows) and compared this with all controls. Indeed, performing such post hoc analyses in our study showed a HR for major osteoporotic fractures of 0.80 (95% CI 0.69–0.92) and a HR for hip fractures of 0.69 (95% CI 0.53–0.91), supporting the assumption that the post hoc results were at least partly caused by bias in the ROSE study.

The screening strategies of ROSE, SCOOP, and SOS were rather similar: All studies were performed in a primary care population of older women; all studies made a preselection of participants in the screening group using a questionnaire before applying bone densitometry; and all three studies used FRAX in risk selection or in treatment thresholds.^12^, ^13^ However, in SOS only participants with ≥1 clinical risk factor for fractures were randomized, whereas in ROSE and SCOOP, all women were randomized. There were small differences in risk selection and treatment thresholds. However, since all three studies seem underpowered, it might be of interest to perform a meta‐analysis.

Some limitations of our SOS study need considerations. First, the study had a limited statistical power. This was caused by the lower proportion of participants with a treatment indication than anticipated. Therefore, we extended the inclusion of participants. The final sample size was restricted by financial means and feasibility. Considering the final number of included participants, this study had a statistical power of 80% to pick up a fracture risk reduction ≥15%, indicating that the study was underpowered to detect a reduction of 9%. This has resulted in wide confidence intervals, so differences in fracture reduction across different fracture types might have been missed. Second, besides the limited participation to screening as well as medication adherence, it should be noted that the data on medication adherence were self‐reported. Self‐reported adherence has been shown to give an overestimation of the actual medication use.^(19)^ A strength of the study was its randomized pragmatic design, which allowed us to study screening in a routine clinical practice. It should be noted that we deviated from the pragmatic nature by providing GPs the option to use a supportive tool for consult notification. Although follow‐up consultations are recommended by the Dutch guidelines, such a tool is not commonly used in usual care. It might have improved treatment adherence. Another strength is that fracture and mortality data were verified in medical records, and the status of persons who dropped out was retrieved as well. This resulted in low numbers of lost to follow‐up.

Screening could be clinically relevant, but the current findings, as well as the findings of the previous two pragmatic screening trials, are inconclusive. The individual studies were not able to show statistical significance of their main outcomes but showed a trend toward fracture reduction, whereas the results on hip fractures were not consistent. Pooled analysis of the results of the three screening studies could provide more insight. The current study showed that important challenges for the implementation of population screening programs are the participation rates, as well as the treatment adherence. Moreover, this study provides suggestions to raise attention for secondary fracture prevention: A large proportion of the participants with an indication for treatment (59%) had a previous fracture. Most of them would already have been offered treatment if they would have been screened in, for instance, a fracture liaison service. Moreover, our explorative finding suggested that screening might be most effective after a recent fracture. These findings support the value of secondary fracture prevention at the community level.

In conclusion, in this study, preselection of high fracture risk followed by bone densitometry and subsequent treatment in primary care did not result in a statistically significant reduction of all fractures. However, considering the dilution in the intervention group as a result of screening and contamination of the control group as a result of usual care, the observed effects on (major) osteoporotic fracture reduction and hip fracture reduction could be clinically relevant. Combined evaluation of the results of the three large randomized pragmatic screening trials is needed to reveal the potential implication of the findings for guidelines and policy makers.

## Disclosures

The authors report a grant from Stichting Achmea Gezondheidszorg and other financial support from Achmea, VGZ Zorgverzekeraar, and Stichting Artsen Laboratorium en Trombosedienst during the conduct of the study. PL declared to have received a lecture fee from Abiogen.
